# Programme theories to describe how different general practitioner service models work in different contexts in or alongside emergency departments (GP-ED): realist evaluation

**DOI:** 10.1136/emermed-2023-213426

**Published:** 2024-04-22

**Authors:** Alison Cooper, Michelle Edwards, Freya Davies, Delyth Price, Pippa Anderson, Andrew Carson-Stevens, Matthew Cooke, Jeremy Dale, Liam Donaldson, Bridie Angela Evans, Barbara Harrington, Julie Hepburn, Peter Hibbert, Thomas C Hughes, Alison Porter, Aloysius Niroshan Siriwardena, Alan Watkins, Helen Snooks, Adrian Edwards

**Affiliations:** 1 Division of Population Medicine, School of Medicine, Cardiff University, Cardiff, UK; 2 Warwick Medical School, University of Warwick, Coventry, UK; 3 School of Hygiene and Tropical Medicine, London, UK; 4 Swansea University Medical School, Swansea University, Swansea, UK; 5 Australian Institute of Health Innovation, Macquarie University, Sydney, New South Wales, Australia; 6 Emergency Department, John Radcliffe Hospital, Oxford, UK; 7 Lincoln School of Health and Social Care, University of Lincoln, Lincoln, UK

**Keywords:** primary care, emergency departments, general practitioners

## Abstract

**Background:**

Addressing increasing patient demand and improving ED patient flow is a key ambition for NHS England. Delivering general practitioner (GP) services in or alongside EDs (GP-ED) was advocated in 2017 for this reason, supported by £100 million (US$130 million) of capital funding. Current evidence shows no overall improvement in addressing demand and reducing waiting times, but considerable variation in how different service models operate, subject to local context.

**Methods:**

We conducted mixed-methods analysis using inductive and deductive approaches for qualitative (observations, interviews) and quantitative data (time series analyses of attendances, reattendances, hospital admissions, length of stay) based on previous research using a purposive sample of 13 GP-ED service models (3 inside-integrated, 4 inside-parallel service, 3 outside-onsite and 3 with no GPs) in England and Wales. We used realist methodology to understand the relationship between contexts, mechanisms and outcomes to develop programme theories about how and why different GP-ED service models work.

**Results:**

GP-ED service models are complex, with variation in scope and scale of the service, influenced by individual, departmental and external factors. Quantitative data were of variable quality: overall, no reduction in attendances and waiting times, a mixed picture for hospital admissions and length of hospital stay. Our programme theories describe how the GP-ED service models operate: inside the ED, integrated with patient flow and general ED demand, with a wider GP role than usual primary care; outside the ED, addressing primary care demand with an experienced streaming nurse facilitating the ‘right patients’ are streamed to the GP; or within the ED as a parallel service with most variability in the level of integration and GP role.

**Conclusion:**

GP-ED services are complex . Our programme theories inform recommendations on how services could be modified in particular contexts to address local demand, or whether alternative healthcare services should be considered.

**WHAT IS ALREADY KNOWN ON THIS TOPIC**

- General practitioners often work in or alongside EDs (GP-ED) with the aim of addressing demand and improving patient flow.
- Available research on whether this aim is achieved is inconsistent with variation in how different service models operate, subject to local context.
- Previously reported qualitative data from our work highlight the complexity in how these service models operate, both within and between GP-ED models. Our quantitative data (of variable quality) did not show a reduction in patient attendances and waiting times with variable findings for hospital admissions and length of hospital stay.

**WHAT THIS STUDY ADDS**

- In this mixed-methods realist evaluation, we present programme theories that describe how the different GP-ED service models operate in different settings.
- Inside the ED, the GP-ED service is integrated with patient flow and general ED demand and GPs take on a wider role than usual primary care. Outside the ED, the service addresses mainly primary care demand, with an experienced streaming nurse facilitating the ‘right patients’ are streamed to the GP. Other models operate within the ED as a parallel service, with most variability in the level of integration and GP role.

**HOW THIS STUDY MIGHT AFFECT RESEARCH, PRACTICE OR POLICY**

- These findings can be applied to local context and allow commissioners and service leads to consider how their GP-ED service could be modified to address local demand, or whether alternative healthcare services should be considered.

## Introduction

Improving ED flow to reduce demand on emergency care services and improve waiting times is a key ambition for NHS England.^1^ However, evidence is needed to inform which healthcare service models best facilitate this.^2^ Establishing primary care general practitioner (GP) services in or alongside EDs was advocated by NHS England pre-pandemic as an approach to manage increasing patient demand, supported in 2017 with £100 million (US$130 million) of capital funding.^3^ This led to an increase in GP-ED service models in England from 81% in 2017 to 95% in 2019, despite limited evidence for effectiveness and safety outcomes.^4–7^

**Figure 1:**
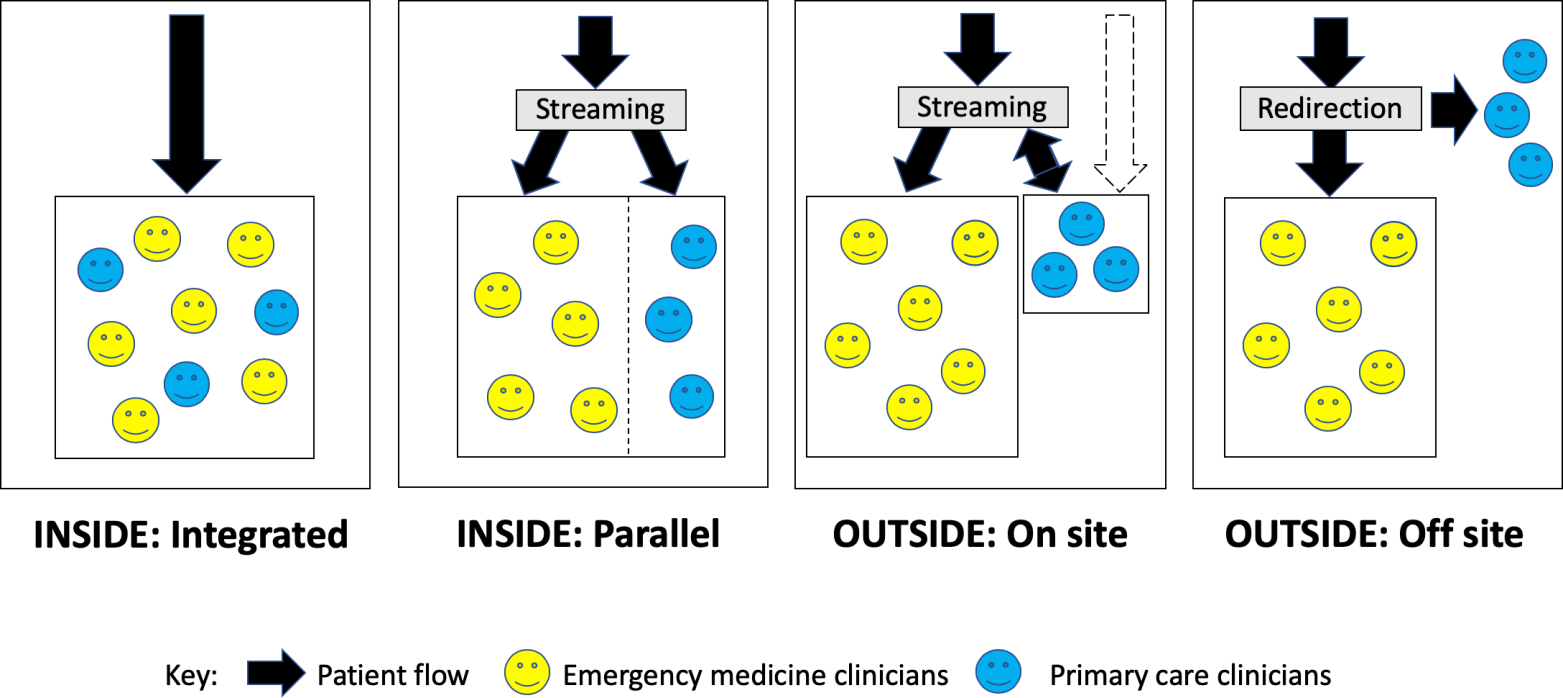
General practitioner (or primary care) service models in or alongside EDs (published as open access in accordance with Creative Commons Attribution 4.0 Unported (CC BY 4.0) license (Cooper *et al*)).^8^

GP-ED (or primary care) service models have been described as operating: **inside** the ED, *integrated* with patient flow or in *parallel* to that activity; or **outside** the ED, *on* or *off the same hospital site* (figure 1).^8^ Using this taxonomy, Benger *et al* conducted a mixed-methods analysis of Hospital Episode Statistics for 32 type 1 English EDs with GP-ED services (6 inside-integrated; 15 inside-parallel; 11 outside-onsite) in England 2017–2019.^9^ They reported slight reductions in the rate of reattendance within 7 days (with negligible clinical significance) but no significant difference in attendance, waiting times, hospital admissions and mortality across all models. However, a substantial degree of heterogeneity was noted in their findings and considerable variation observed during qualitative data collection at their 10 case study sites for how the service models operate, subject to local context.^9^

Evaluation of the effectiveness of these service models that work differently in complex adaptive socio-technical systems that vary in location, population demographic, workforce skillset and wider service provision is challenging.^10^ Routinely collected data may not enable detailed understanding of the complexity, and analysis and interpretation may be limited by variable quality of those data.^11^ Realist methods are well suited to evaluating complex interventions such as these, exploring variation and nuance in different contexts to explain ***what works, for whom, under what circumstances and how***.^12^ Realist methods generate ‘programme theories’ from ‘initial rough’ and ‘refined’ theories, described by context-mechanism-outcome (CMO) configurations. These provide a means to understand the interactions that lead to outcomes of interest, and which can be a basis for making changes to improve those outcomes.^12^

In this paper, we integrate the findings of our mixed-methods realist evaluation the GPs in EDs study (2017–2021) including 13 GP-ED case study sites from England and Wales (3 hospitals with inside-integrated; 4 with inside-parallel; 3 with outside-onsite; and 3 control models (no GP-ED) in operation).^13^ (Key findings from the 17 publications are listed in online supplemental appendix 1.) We present programme theories to describe how the different GP-ED models operate and are influenced by wider system, department and individual factors. These provide more nuanced interpretation, according to each GP-ED model used, than is captured in the taxonomy of models.^8^ Commissioners and clinical leads can then consider further how their services are configured for their aims and local context or which factors, in particular the mechanisms of how models are operating, may be modifiable to address local demand.

10.1136/emermed-2023-213426.supp1 Supplementary data

## Methods

### Study design

Realist methodology is a theory-driven approach that identifies mechanisms (M) that explain how or why contexts (C) relate to outcomes (O), describing theories as CMO configurations; definitions in table 1.^12^ Programme theories were developed through refining initial rough theories from a rapid realist review,^6^ and analysis of national patient safety incident reports,^14^ with previously collected and analysed qualitative and quantitative case site data.^13^ We followed RAMESES reporting and publication standards (online supplemental appendix 2).^15^

**Table 1:** Realist methodology definitions^12^

Context (C)	Pre-existing conditions which influence the success or failure of different interventions or programmes
Mechanism (M)	Characteristics of the intervention and people’s reaction to it; how it influences their reasoning
Outcome (O)	Intended and unintended results of the intervention as a result of a mechanism operating within a context
Initial rough theory	An early theory, informed by available evidence, about how, why for whom, in what circumstances the intervention is thought to work described as a context-mechanism-outcome configuration
Refined theory	An initial theory that has been refined using primary or secondary evidence
Programme theory	An overall high-level theory summarising how the intervention works, developed using the theories refined from the data

### Ethical approval

The fieldwork for case study site visits, local patient safety incident report analysis and staff and patient interviews were carried out after ethical approval from the Wales Research Ethics Committee on 23/07/2017 (ref 17/WA/0328).

### Summary of previous work

### Case site selection

Details of this work were previously reported.^13^ Briefly, this work began in 2017 when we recruited a purposive sample of ‘case sites’ from a national survey sent to the Clinical Directors of all Type 1 Emergency Departments (consultant-led 24 hours services with full resuscitation facilities) in England and Wales, with follow-up key informant telephone interviews.^16^ The included sample of 13 case sites, with characteristics listed in online supplemental appendix 3, included:

- Three inside-integrated models.
- Four inside-parallel models (one was reclassified following the visit).
- Three outside-onsite models (outside the ED, on the same hospital site).
- Three sites with no GPs.^13^

### Qualitative data collection and analysis

As previously reported, two researchers visited all sites with a GP service (n=10; 2–4 days) and sites with no GPs for 1 day (n=3; 1 day) between January 2018 and April 2019.^13^ We used initial rough theories generated from the rapid realist review, for example, if GPs maintain their usual approach when working in EDs, investigation use and process times could reduce,^6^ to develop realist interview guides for theory testing and refining to explore ***how*** GPs maintain their usual GP approach in ED settings (online supplemental appendix 4).^17^ We requested local patient safety incident reports related to the GP-ED model^14^ and invited patients presenting with marker conditions (online supplemental appendix 5) for interviews to describe their experiences when GPs work in EDs.^18^ We analysed interview and observation data from multiple sources and applied knowledge from conceptual frameworks and formal theories to refine our initial theories. We then mapped CMO configurations against different GP-ED models to compare across different types of service, to describe model-related mechanisms that contribute to outcomes such as GP approach, use of investigations, process times and patient experience. We presented findings at a national event in December 2019 (n=70 attendees) for stakeholder feedback.

### Quantitative data collection and analysis

Patient-level routinely collected data relating to ED attendances and subsequent hospital admissions were obtained from Hospital Episode Statistics Accident and Emergency and Admitted Patient Care datasets (via NHS Digital) for study sites located in England and from Emergency Department Data Set and Patient Episode Database for Wales (via SAIL) for study sites located in Wales as previously reported.^13^ The attendance-level data were summarised as time series (per site, aggregating data for each study fortnight) for the following variables:

- Counts of ED attendances.
- Reattendance at same ED.
- ED attendance leading to a hospital admission (patient record in APC dataset).
- Investigations undertaken during ED attendance.
- Treatments delivered during ED attendance.
- Average time (minutes) of ED attendance.
- Length of stay (days) of hospital admission.

For all variables, we used standard time series analysis methods to assess the nature and extent of linear trends and seasonality in data before and after introduction of a GP-ED model at those sites.^13^

### Current analysis: Mixed-methods synthesis and programme theory development

For this paper, we conducted a mixed-methods synthesis with two separate approaches to develop our programme theories. In approach 1, we further tested and refined our theories developed through qualitative data analysis with outcome data from our quantitative analysis (see table 2).^12^ For example, for GP-ED models where GPs maintain their usual approach in ED settings, did process times and investigation use reduce? In approach 2, we identified findings for key outcomes from the statistical analysis and cross-checked with the qualitative dataset (see table 3) to ensure we used both inductive and deductive approaches to maximise insights generated.^19 20^ Findings were used to develop programme theories describing high level individual, department level and wider system influences on the function of the different GP-ED models.^12^

**Table 2:** Mixed-methods analysis approach 1—key insights from qualitative case site data analysis, explored through quantitative data analyses^13^

Theme	Key insights from qualitative data analysis^13^	Explored with quantitative data^13^
Streaming and flow	Experienced staff make competent and confident decisions to direct patients to the most appropriate clinician/pathway, optimising patient flow through the ED. When EDs are short staffed or experience ‘exit block’, streaming will not improve patient flow through the ED.	Data on waiting time to treatment were not available and were generally poorly and inconsistently recorded. However, there was an upward trend in the length of time in the department for all patients, (those seen by ED staff and GP-ED service models) at most study sites (including the three control sites) over the study period both pre and post intervention. Two sites showed a statistically significant decrease in time in the department post intervention, both of which (parallel-2, outside-3) we had identified from qualitative data as having more features of effective streaming. Outside-3, however, also had a separate unit for geriatric emergency care which may have influenced length of stay in the ED. The other sites where we categorised streaming as appearing most effective (integrated-1, outside-1), showed a trend towards increase in the length of time all patients spent in the department. Another site (parallel-3) which had also established a system for redirecting patients to community primary care services also showed a non-significant increase in time in the ED after the GP model was introduced.
GP role	Experienced GPs, confident in their clinical skills and with awareness of primary care resources, with a clearly defined ‘GP role’ in the ED, can use their usual ‘GP approach’ (no acute investigations or observation time) for patients presenting with primary care type conditions to reduce patient time in the ED and acute hospital admissions. However, if there is an expectation for GPs to use acute investigations and ED protocols/governance processes, then GPs may adopt an emergency medicine approach with no change in time in the department or hospital admission rates.	There was a positive trend for increasing time in the department over time for all three control sites, and most GP-ED model sites. Time in the department showed a trend towards increasing both pre and post intervention at the two sites of interest where the GP role (maintain usual GP approach) appeared well supported (parallel-4 and outside-1), no statistically significant change post intervention. Investigation data were non-stationary suggesting poor quality and that rates were influenced by coding/recording practices. Findings were a mixed picture of the use of investigations across control sites and all GP-ED models. There were three sites where the GPs had no access to acute investigations (parallel-4, outside-1, outside-2) where we expected the ‘usual GP approach’ to be enabled, one site (parallel-4) showed a statistically significant change in trend from increasing investigations (pre intervention) to decreasing investigations (post intervention). Data quality at outside-2 precluded analysis. For acute hospital admissions, there were increasing admissions over time for all the control sites and a mixed picture across the intervention sites. Changes in trend were largely towards fewer admissions. Two changes reached statistical significance. At parallel-4 (identified as an example of a site facilitating ‘the GP role’) there was a reversal in trend from increasing to decreasing admissions post intervention. At integrated-1, where a more emergency medicine role would be expected there was an increase on the background upward trend in admissions post intervention.
Patient experience	If a patient attends the ED with a problem that is dealt with in a timely appropriate manner, then this is seen as acceptable.	Our routine data did not identify any model which was consistently associated with shorter stays in the ED; in fact, overall time in the department increased at most sites over the study period. We did not have any data quantifying patient satisfaction across the different sites to test our theory of a relationship between waiting times and satisfaction.
Impact on patient attendances with non-urgent conditions	Distinct urgent primary care services may offer convenient access to primary care resulting in ‘provider-induced demand’.	Attendance rates showed a mixed picture across control sites, across all intervention sites and within GP-ED models, but with a general picture of attendances increasing over time. At one of the ‘highly visible’ sites of interest (outside-3) this reached statistical significance, with statistically significant increases post intervention also seen at two other sites (integrated-1, parallel-1). However, the quantitative data measured all ED attendances (with no focus on primary care attendances) and the qualitative data described staff perceptions based on their experiences of individual patients attending with primary care needs. Reattendances were measured at 28 days. There was a trend demonstrating increased reattendances for most control sites and intervention sites. All four parallel sites demonstrated a downward trend pre intervention, with a subsequent increase in reattendances post intervention (3 of these reached statistical significance including parallel-4, a site of particular interest for facilitating the GP role). Data quality at outside-1 prevented analysis.

Reproduced with permission from Davies et al, NIHR Journals Library. This is an Open Access publication distributed under the terms of the Creative Commons Attribution CC BY 4.0 licence, which permits unrestricted use, distribution, reproduction and adaptation in any medium and for any purpose provided that it is properly attributed. See: https://creativecommons.org/licenses/by/4.0/.^13^

GP, general practitioner.

**Table 3:** Mixed-methods analysis approach 2—findings for key outcomes from the statistical analysis, triangulated with qualitative findings^13^

Outcome measured	Key findings from the statistical analysis^13^	Explored with the qualitative data^13^
ED attendances over time (2010–2018)	No evidence of reduction in attendances, with increased attendances in all intervention sites where change could be assessed with confidence (some statistically significant).	Perception at some sites that increases in primary care demand have been triggered by the visibility, accessibility and local awareness of the GP-ED model.
ED reattendances within 28 days	Controls all show increases over time. At intervention sites, all that could be calculated show increased reattendances post intervention except for integrated-2 (statistically significant decrease).	Integrated-2 was a very small ED and saw a limited range of patients with more unwell patients being taken to an alternative hospital. (After the study period this ED was downgraded to an urgent care centre.)
Average time in the ED	Increased at all 3 control sites during study period and most intervention sites. Two intervention sites (outside-3, parallel-2) identified with evidence of reversal in upward trends in average time in the ED.	There was a new frailty unit at outside-3 introduced at the same time as the GP-ED model. Note this site also showed a negative trend for admissions.
Investigation use	Mixed picture of investigation use from control sites (flat or upward trends). Also, a mixed picture within each model. Two sites of interest showing statistically significant decreases in investigations post intervention (parallel-3, parallel-4).	At parallel-4 GPs had no access to investigations. At parallel-3 there was a structured pathway for redirecting patients to community primary care. GP-ED model saw only small proportion of overall ED attendances limiting potential impact.
Admissions	All 3 control sites have upward trends, mixed picture across intervention sites. Statistically significant post intervention changes identified at two sites. Parallel-4 showed a reversal of direction of trend from increasing to decreasing admissions. Integrated-1 showed increased admissions post intervention.	At parallel-4, ‘the GP role’ was supported and staff gave examples of GPs managing paediatric patients without the need for admission. At integrated-1, staff perceived that demand was increasing due to new housing developments in the area.

Reproduced with permission from Davies et al, NIHR Journals Library. This is an Open Access publication distributed under the terms of the Creative Commons Attribution CC BY 4.0 licence, which permits unrestricted use, distribution, reproduction and adaptation in any medium and for any purpose provided that it is properly attributed. See: https://creativecommons.org/licenses/by/4.0/.

GP, general practitioner.

### Patient and public involvement

Patients and public members (BH, JH) were involved in the study design, as coapplicants in the funded study and are coauthors on this publication.^13^ They used their experience as NHS patients to contribute to this research, reflecting on interview transcripts and findings. They supported recruitment and involvement of public and patient contributors to the stakeholder events. They were involved in discussing the draft data and have a publication reflecting their experiences in this study.^21^

## Results

Qualitative data collection at case study sites showed considerable variation between and within GP-ED models. Our published papers describe how: integration of GPs with the ED team (and resources, eg, IT systems) varied^16^; services varied in allocating (or streaming) patients with different presenting complaints to the GP service^22^; GP role and interprofessional communication varied with some services encouraging a ‘usual GP approach’ with minimal access to acute investigations or opportunity to observe patients, and others expecting GPs to use ED facilities^23^; some GPs took on roles supervising junior ED clinicians, including allied health professionals^13^; employment, contractual and governance status of GPs varied, and influenced flexibility in deploying GP and ED team members to times or places of greatest need^24^; there was concern that highly visible services may attract additional ‘provider-induced’ demand^25^; and influence patients’ expectations and experiences of using the service.^26^ Quantitative data (2010–2018) from the case study sites (accessed via NHS Digital and SAIL Databank) were of variable quality,^13^ but overall there was no GP-ED model found to be superior. GP-ED service models did not reduce attendances and waiting times (which increased across time regardless of GP-ED model (or none) used), with a mixed picture for hospital admissions and length of hospital stay.^13^

### Mixed-methods synthesis

Tables 2 and 3 outline our mixed-methods findings across the qualitative and quantitative analyses. Due to poor quantitative data quality, links between context and mechanisms from qualitative data where were often difficult to refine with quantitative outcome data. Analysis at ‘outside-onsite’ sites, where GPs generally maintained a GP role, were limited by poor quantitative data quality. Our qualitative data offered some explanation for some patterns observed in the routine data analysis which included wider system factors such as a frailty unit potentially influencing waiting times at one case site (outside-3) and a new housing estate contributing to increasing hospital admissions at another (inside-integrated-1).

### Programme theories

**Figure 2:**
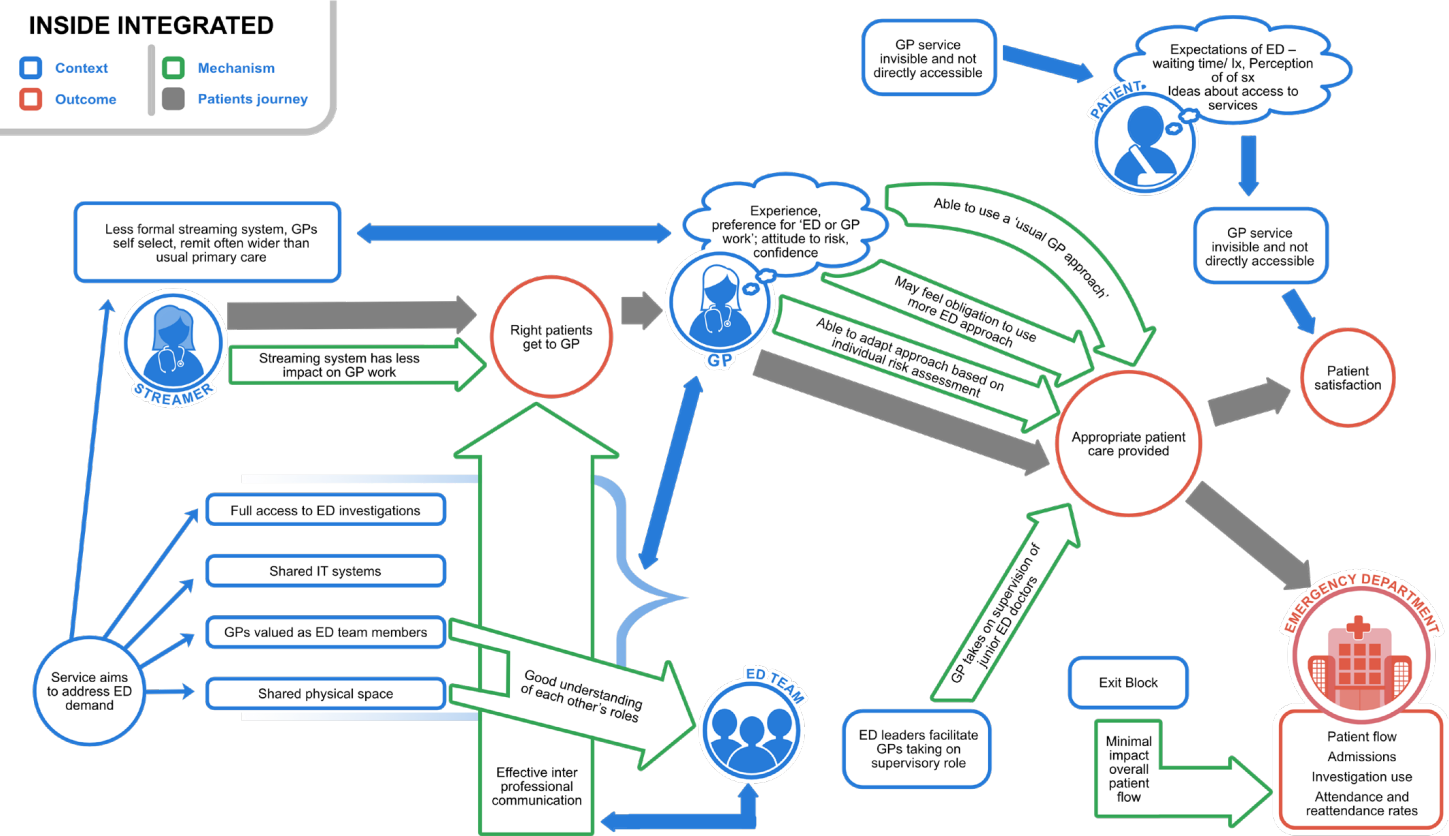
Programme theory to describe contexts, mechanisms and outcomes in inside-integrated general practitioner services in or alongside EDs (GP-EDs). Models where the streaming process is less influential, GPs may take on an ED clinician role and the ‘invisibility’ of the GP service limits patient expectation.Reproduced with permission from Davies et al. NIHR Journals 13

**Figure 3:**
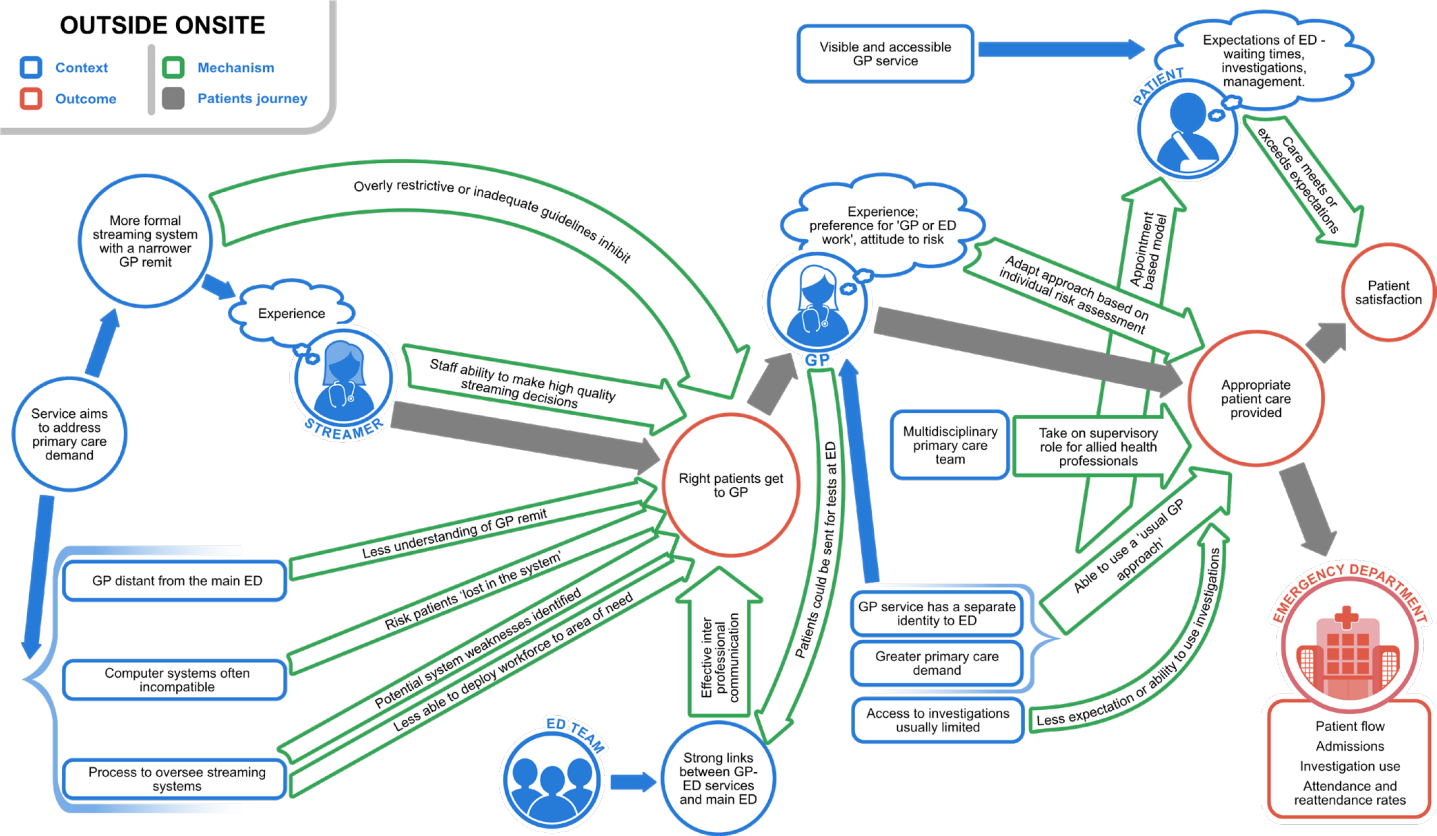
Programme theory to describe contexts, mechanisms and outcomes in outside-onsite general practitioner services in or alongside EDs (GP-EDs). Models with greater complexity ensuring the right patients saw the GP and high visibility of the service having greater impact on patient expectation.Reproduced with permission from Davies et al. NIHR Journals 13

**Figure 4:**
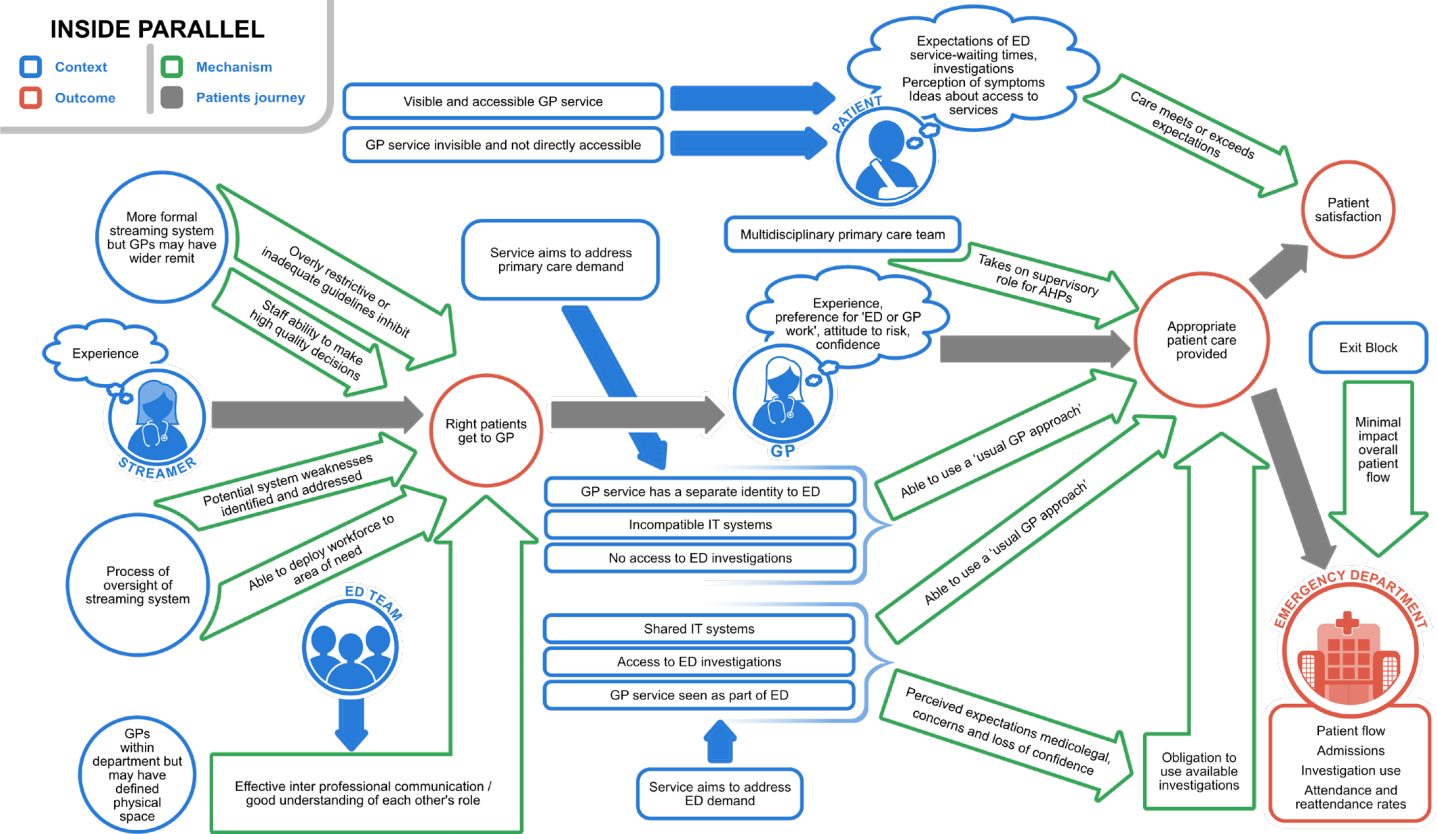
Programme theory to describe contexts, mechanisms and outcomes in inside-parallel general practitioner services in or alongside EDs (GP-EDs). Models with the most variation in service set up and lack of clarity on GP role.Reproduced with permission from Davies *et al*. NIHR Journals 13

We focused our programme theories on highlighting how the differences in actions (largely at an individual clinician level, both within and between the GP-ED models in different settings) influenced how the three GP-ED models worked. These theories have generally been refined from the qualitative data due to the quality of routine (quantitative) data available for analysis.^13^ Figures 2–4 present high-level summaries of key features for each programme theory of the GP-ED models described: inside-integrated, inside-parallel, outside-onsite. In each, the patient journey is depicted with grey arrows from being selected to see a GP, receiving care (±investigation and/or treatment) and resulting in patient experience and flow (outcomes) through the department. Context is labelled in blue, mechanisms within the green arrows and outcomes in red circles.^13^

### Inside-integrated sites

These services aimed to address general ED demand (rather than primary care demand) and were often funded from within the same NHS Trust as the ED. The GPs worked in the same physical space as ED staff with shared IT systems, full access to acute investigations and were valued as members of the ED team. Compared with the other models, the streaming process was less influential with effective interprofessional communication and good understanding of each other’s roles facilitating the right patient to be seen by the GP. GPs also self-selected patients that fitted their own clinical remit, which was often wider than primary care type problems. GP experience, confidence, preferences and attitudes to risk, along with ED leadership support, were described to influence whether GPs treated patients with a GP approach or adopted an emergency medicine approach. Unlike in other models, inside-integrated models allowed for GPs to take on supervision of junior ED doctors which was considered a particular asset at some sites. The ‘invisibility’ of the GP (from the ED) service meant its impact on patient expectations was also more limited (figure 2).^13^

### Outside-onsite sites

At outside-onsite sites, quantitative data quality was poorer, with one site excluded from analysis due to poor data quality. Outside-onsite models typically aimed to deal specifically with primary care demand and had a clearly defined identity in a separate physical location, usually with incompatible IT systems to the ED. As a result, there were usually agreed criteria and pathways for accessing the service and GPs’ remit generally remained similar to that of community primary care (without access to ED investigations). Qualitative data highlighted the process of ensuring the right patient could see the GP was more complex with potential for patients to get ‘lost’ between systems or inappropriate patients being referred to the GP service. An experienced streaming nurse with clear guidance and good communication with the ED team was described to mitigate this. The high visibility and accessibility of these services were likely to have a greater impact on patients’ expectations and experiences. At these sites GPs sometimes took on a supervisory role for a wider primary care team including, for example, nurse practitioners and paramedics (figure 3).^13^

### Inside-parallel models

Inside-parallel models showed the most variation in the way the services were set up. Some were more similar to inside-integrated models, others more distinct as inside-parallel models. The level of primary care demand (and aim of addressing this) varied at these sites and perceptions differed about how integrated the GP service and the ED were intended to be at different sites. Some services had shared IT systems, in others the IT services were incompatible. While streaming guidelines were often formalised, there was sometimes flexibility for GPs to take on a wider (emergency medicine) role, with variable access to ED level investigations, and to allow for personal characteristics of individual clinicians, including preferred ways of working, experience, confidence levels and medico-legal concerns to shape their case-mix. Techniques described by GPs as ways to mitigate risks when using the ‘GP approach’ to treat higher risk ED patients included longer consultations, different thresholds for investigation or admission, and developing supplementary emergency medicine skills. The lack of clarity around the breadth of the GP role could be a particular concern for sites with this model, although this could be overcome in settings with strong clinical ED leadership and effective interprofessional communication to facilitate understanding of each other’s roles. The visibility and accessibility of the GP service was described to influence patient expectations and satisfaction with the service (figure 4).^13^

## Discussion

### Main findings

Our study shows the complexity in these service models with variation in the scope of the GP role and scale of the service, both within and between GP-ED models, and other external influences, all of which present challenges in evaluation. Quantitative data were of variable quality (poorer quality for outside models) but overall there was no reduction in attendances and waiting times, and a mixed picture for hospital admissions and length of hospital stay. We present programme theories to describe how three previously described GP-ED service models operate: inside the ED, integrated with patient flow largely addressing general ED demand with GPs requiring a wider skillset than usual primary care and the streaming process less influential; outside the ED addressing primary care demand in a separate service with an experienced streaming nurse facilitating the ‘right patients’ are streamed to the GP; or within the ED as a parallel service with most variability in the level of integration (including IT services, investigation access) and GP role (primary care vs emergency medicine), facilitated by strong clinical ED leadership and interprofessional communication.^8 13^

### Strengths and limitations

The analysis was strengthened by the 13 case study sites, purposively recruited for theory testing and refinement with different service models in different sized hospitals, geographically spread across England and Wales. Researchers applied a consistent realist approach, with initial rough theories developed from the literature tested through qualitative methods.^17^ Routinely collected quantitative data were available for analysis at most case study sites.

Limitations include all data being collected prior to the COVID-19 pandemic. Since then, other service models for accessing care, such as ‘call first’ for walk-in patients, have been introduced in EDs for patients with primary care type problems.^27^ There has also been increasing interest in the roles of the non-medical workforce in the urgent and emergency care system, with evaluation in progress.^28^ However, evidence to inform how to manage increasing patient demand following the pandemic is still relevant. We did not identify any case sites where GPs screened patients at the front door in a *gatekeeper role*, although there may be departments operating this service model of which we were unaware.^6^ The visits, at 3 days were short, limiting data collection; there was especially low recruitment for patients for interview.^18^ Qualitative data are subject to researcher and participant perceptions. Quantitative data availability, extraction and analysis were delayed due to pandemic constraints.

### Context of current literature

Increasing demand on UK EDs is multifactorial including an ageing society with increasing multimorbidity,^29^ a reduction in the number of community GPs coping with an increasing workload,^30^ inadequate social care preventing hospital discharge for medically fit patients causing ‘exit block’^31^ and the impact on the general health and social care workforce following the COVID-19 pandemic and Brexit.^32^

Internationally, GP-ED service models have been introduced to address increasing ED attendances.^6 7^ Our findings highlight the challenges in evaluating the effectiveness of these service models when data quality is variable and they are part of complex adaptive socio-technical systems.^10^ High-level routine data analysis of Hospital Episode Statistics may not be sufficient to understand the nuanced complexity of the individual EDs when aggregated to national level. Our findings integrate a large programme of work including 17 published papers (see online supplemental appendix 1)^13^ and are consistent with findings by Benger *et al*,^9^ that GPs appear less influential in these overall complex processes than patient and wider system factors. The impact of GP-ED models on the community primary care workforce is unknown, as also whether other integrated healthcare and social care service models such as helping discharge medically fit patients back into the community, may have more impact on ED patient flow.^31^

### Recommendations for policy and practice

- Commissioners and service providers should recognise the complexity of system, department and individual patient and staff factors, that can influence how GP-ED service models operate.
- The *aim* of service provision (eg, to address primary or emergency demand or staff recruitment needs), the role of the GPs within the service and the most appropriate GP-ED model should be clarified, based on local need.
- The existing service model may be modified accordingly, for example, developing (or not) a separate area for primary care provision, using an experienced streaming nurse to ensure the right patients get to the GP, which role (GP or emergency clinician, including access to investigations) the GP is supported to adopt in that ED, encouraging interprofessional communications to improve understanding of the GP role and remit (and levels of GPs’ of experience to provide such ‘more primary care’ or ‘more emergency medicine’ roles), integration of IT systems, governance of employed GP staff; or whether an alternative healthcare service may be considered to meet local demand.
- Quantitative data quality needs to be improved across primary and secondary care services to enable future evaluation of similar GP-ED services.

### Further research

Further research is needed to determine the impact of GP-ED service models on sustainability of the community primary care workforce. With few distinct effects of GP-ED models noted for our key quantitative outcomes, cost-effectiveness analysis was not warranted. More nuanced resource use implications for the different models in relation to their costs and consequences in different contexts may be valuable to guide future service design.

### Conclusion

GP-ED services are complex and influenced by individual, department and wider system influences. Our programme theories describe how the different service models operate in different settings, to inform recommendations about how services could be modified in particular contexts to address local demand, or whether alternative healthcare services should be considered.

## Data Availability

Data are available upon reasonable request. The full NIHR peer-reviewed report is available at Davies F, Edwards M, *et al.* Evaluation of different models of general practitioners working in or alongside emergency departments: a mixed methods realist evaluation. *NIHR Journals Health and Social Care Delivery Research* (in press) https://www.journalslibrary.nihr.ac.uk/programmes/hsdr/1514504/%23/.
