# Supplementary material for: Programme theories to describe how different general practitioner service models work in different contexts in or alongside emergency departments (GP-ED): realist evaluation
Source: Emerg Med J. 2024 Apr 22;41(5):287–95. doi: 10.1136/emermed-2023-213426 (PMC11041563; doi:10.1136/emermed-2023-213426)
Supplement: Supplementary data [file emermed-2023-213426supp001.pdf]

Appendix 1: Publications resulting from the GPs in EDs Study (NIHR HS&DR: 1514504)

Journal and summary	Reference	Key findings
**NIHR Journals Library** (in press 2023)<br><br>NIHR Peer reviewed Final Report	Davies F, Edwards M, Price D et al. Evaluation of different models of general practitioners working in or alongside emergency departments: a mixed methods realist evaluation Health and Social Care Delivery Research (in press) https://www.journalslibrary.nihr.ac.uk/programmes/hsdr/1514504/#/	GPs commonly work in EDs but delivery models vary widely in terms of the scope of the GP role and the scale of the GP service. Routine data were of variable quality, limiting our analysis. Time series analysis demonstrated trends across intervention sites for: increased time spent in the ED; increased ED attendances and reattendances; and mixed results for hospital admissions. Evidence on patient experience was limited but broadly supportive; we identified department level processes to optimise the safety of GP-ED models and present a programme theory as principal output of the study to describe how these service models were observed to operate
**BMJ Open** (2019)<br><br>How and why GP-ED models work, a review of the literature	Cooper A, Davies F, Edwards M, Anderson P, Carson-Stevens A, Cooke MW, et al. The impact of general practitioners working in or alongside emergency departments: A rapid realist review. BMJ Open. 2019;9(4):1–11.	Multiple factors influence the effectiveness of different service models. Further research is needed.
**EMJ** (2019)	Cooper A, Edwards M, Brandling J, Cooke M, Davies F, Hughes T, et al. Taxonomy of the form and function of primary care services in	A spectrum of integration identifies constructs that influence how services function – from being closer to an emergency medicine

A taxonomy of the form and function of GP-ED models	or alongside emergency departments: concepts paper. EMJ. 2019;36:625–30	service, to usual primary care – as a basis for future evaluation of service models.
**BMJ** (2020)<br><br>Evidence for the effectiveness of streaming ED patients to primary care services	Cooper A, Carson-Stevens A, Hughes T, Edwards A. Is streaming patients in emergency departments to primary care services effective and safe? BMJ 2020;368:8–11	There is limited, outdated evidence to show whether streaming patients to primary care services improves patient flow and reduces cost, and evidence for patient safety outcomes is lacking
**Health Policy** (2021)<br><br>How GP-ED funding mechanisms influence wider system outcomes.	Choudhry M, Edwards M, Cooper, A et al. Senior clinical and business manages' perspectives on the influence of different funding mechanisms, and barriers and enablers, to implementing models of employing General Practitioners in or alongside emergency departments: Qualitative study Health Policy 2021;125(4)482-488	Leaders caution against the use of private providers.
**BMC Emergency medicine** (2020)<br><br>Clinical leaders' experiences of implementing GP-ED models	Edwards M, Cooper A, Davies F et al. Emergency department clinical leads' experiences of implementing primary care services where GPs work in or alongside emergency departments in the UK: qualitative study BMC Emerg Med 20, 62(2020)	Different models of service are necessary based on local contextual circumstances.
**BMC Medical Research Methodology** (2020)	Price D, Edwards M, Carson-Stevens A, Cooper A, Davies F, Evans B, et al. Challenges of recruiting emergency department patients	The key factor hindering patient recruitment was a lack of face-to-face contact between patients and researchers.

Challenges of recruiting ED patients to qualitative research	to a qualitative study: a thematic analysis of researchers' experiences. BMC Med Res Methodol. 2020;20(1):151	
**BMC Emergency Medicine** (2019)<br><br>Diagnostic error in the ED – learning from patient safety incident reports.	Hussain, F., Cooper, A., Carson-Stevens, A. *et al.* Diagnostic error in the emergency department: learning from national patient safety incident report analysis. *BMC Emerg Med* 19, 77 (2019)	System modifications are needed to provide better support in patient assessment and investigation interpretation
**IJPDS** (2020)<br><br>Key approaches to successful PPI in primary and emergency care research.	Evans B, Wallace C, Snooks H et al. "Public involvement and engagement in primary and emergency care research: the story from PRIME Centre Wales", *International Journal of Population Data Science*, (2020)5(3) doi: 10.23889/ijpds.v5i3.1363	A supportive context, resources and stable research environment enable public involvement and engagement to be embedded throughout the research cycle
**Emergency Nursing**<br><br>A classification of primary care streaming pathways in UK emergency departments	Edwards M, Cooper A, Thomas H et al. A classification of primary care streaming pathways in UK emergency departments: Findings from a multi-methods study comprising cross-sectional survey; site visits with observations, semi-structured and informal interviews. International Emergency Nursing 2021;56:10100	Local clinical leaders and managers need to consider which pathway(s) may best suit their local contexts and needs.
**BJGP** (2021)<br><br>Identifying safe care processes when GPs	Cooper A, Carson-Stevens A, Edwards M, Davies F, Donaldson LJ, Anderson P, et al. Identifying safe care processes when GPs work	Experienced streaming nurses using local guidance and early warning scores, clear governance to support GP decision making,

work in or alongside emergency departments	in or alongside emergency departments: A realist evaluation. British Journal of General Practice. 2021;71(713)	and strong clinical leadership to promote teamwork and communication were perceived contributors to safe patient care
**EMJ**<br><br>Provision of GP services in or alongside EDs in England	Brant H, Voss S, Morton, K et al. Current provision of general practitioner services in or alongside emergency departments in England. EMJ 2021;0:1-4	The majority of EDs in England have adopted a GP service. The popularity of different models is dependent on the availability of capital funding to finance structural changes.
**BMC Emergency medicine**<br><br>Learning from diagnostic errors to improve patient safety when GPs work in or alongside EDs	Cooper A, Carson-Stevens A, Cooke M, Hibbert P, Hughes T, Hussain F, Siriwardena N, Snooks H, Donaldson L EA. Learning from diagnostic errors to improve patient safety when GPs work in or alongside emergency departments: incorporating realist methodology into patient safety incident report analysis." *BMC emergency medicine* 2021;21(1): 1-13	Potential priority areas for improvement are a standardisation of initial assessment; clinical decision support for high-risk conditions; and standardised communication between services
**BMC Emergency Medicine**<br><br>Patients experiences of attending EDs with primary care services	Price D, Edwards M, Davies F, Cooper A, McFadzean J, Carson-Stevens A, et al. Patients' experiences of attending emergency departments where primary care services are located: qualitative findings from patient and clinician interviews from a realist evaluation. BMC Emerg Med. 2022;22(1):12	Patients generally find being streamed to a primary care service acceptable if they receive suitable treatment in a timely manner, with clear communication from staff
**Emergency nursing**<br><br>Effectiveness of streaming on decision-making,	Edwards M, Cooper A, Hughes T, Davies F, Price D, Anderson P, et al. The effectiveness of primary care streaming in emergency departments on decision-making and patient flow and safety – A realist evaluation. Int Emerg Nurs. 2022;62	Services should: be designed in line with local circumstances and staff skill; use trained streaming nurses; monitor business to help

patient safety and patient flow		waiting time and flow; and involve staff in developing streaming guidance.
**Health expectations** (2022)<br><br>Implementing public involvement throughout the research process	Evans B, Carson-Stevens A, Cooper A et al. Implementing public involvement throughout the research process—Experience and learning from the GPs in EDs study. Health Expectations. 2022;25(5)	Public involvement was more extensive and detailed than envisaged. Public contributors played an active role throughout the research progress, including key involvement in data collection, analysis and dissemination
**BMC Emergency Medicine** (2022)<br><br>Provider Induced Demand	McFadzean IJ, Edwards M, Davies F, Cooper A, Price D, Carson-Stevens A, et al. Realist analysis of whether emergency departments with primary care services generate 'provider-induced demand.' BMC Emerg Med 22, 155 (2022)	EDs with distinct primary care services were perceived to attract demand for primary care because services were visible, known or enabled direct access to health care services. Other influencing factors included patients' experiences of accessing primary care, community care capacity, service design and population characteristics.

Appendix 2: RAMESES reporting standards for realist evaluation

Item to be included	Page number/Section
TITLE	
1. In the title, identify the document as a realist evaluation.	1
SUMMARY OR ABSTRACT	
2. Journal articles will usually require an abstract, while reports and other forms of publication will usually benefit from a short summary. The abstract or summary should include brief details on: the policy, programme or initiative under evaluation; programme setting; purpose of the evaluation; evaluation question(s) and/or objective(s); evaluation strategy; data collection, documentation and analysis methods; key findings and conclusions. Sufficient detail should be provided to identify that a realist approach was used and that realist programme theory was developed and/or refined .	3
INTRODUCTION	
3. Rationale for evaluation. Explain the purpose of the evaluation and the implications for its focus and design.	5
4. Programme theory. Describe the initial programme theory (or theories) that underpin the programme, policy or initiative.	6
5. Evaluation questions, objectives and focus. State the evaluation question(s) and specify the objectives for the evaluation. Describe whether and how the programme theory was used to define the scope and focus of the evaluation.	6
6. Ethical approval. State whether the realist evaluation required and has gained ethical approval from the relevant authorities, providing details as appropriate. If ethical approval was deemed unnecessary, explain why.	8
METHODS	
7. Rationale for using realist evaluation. Explain why a realist evaluation approach was chosen and (if relevant) adapted	5-6
8. Environment surrounding the evaluation. Describe the environment in which the evaluation took place	4
9. Describe the programme policy, initiative or product evaluated. Provide relevant details on the programme, policy or initiative evaluated	4
10. Describe and justify the evaluation design. A description and justification of the evaluation design (i.e. the account of what was planned, done and why) should be included, at least in summary form or as an appendix, in the document which presents the main findings. If this is not done, the omission should be justified and a reference or link to the evaluation design given. It may also be useful to publish or make freely available (e.g. online on a website) any original evaluation design document or protocol, where they exist	7-8
11. Data collection methods Describe and justify the data collection methods – which ones were used, why and how they fed into developing, supporting, refuting or refining programme theory. Provide details of the steps taken to enhance the trustworthiness of data collection and documentation.	8

12. Recruitment process and sampling strategy. Describe how respondents to the evaluation were recruited or engaged and how the sample contributed to the development, support, refutation or refinement of programme theory<br>13. Data analysis. Describe in detail how data were analysed. This section should include information on the constructs that were identified, the process of analysis, how the programme theory was further developed, supported, refuted and refined, and (where relevant) how analysis changed as the evaluation unfolded	8-9<br>8-10
RESULTS<br>14. Details of participants. Report (if applicable) who took part in the evaluation, the details of the data they provided and how the data was used to develop, support, refute or refine programme theory.<br>15. Main findings. Present the key findings, linking them to contexts, mechanisms and outcome configurations. Show how they were used to further develop, test or refine the programme theory	8-17<br>11-17
DISCUSSION<br>16. Summary of findings. Summarise the main findings with attention to the evaluation questions, purpose of the evaluation, programme theory and intended audience<br>17. Strengths, limitations and future directions. Discuss both the strengths of the evaluation and its limitations. These should include (but need not be limited to): (1) consideration of all the steps in the evaluation processes; and (2) comment on the adequacy, trustworthiness and value of the explanatory insights which emerged. In many evaluations, there will be an expectation to provide guidance on future directions for the programme, policy or initiative, its implementation and/or design. The particular implications arising from the realist nature of the findings should be reflected in these discussions<br>18. Comparison with existing literature. Where appropriate, compare and contrast the evaluation's findings with the existing literature on similar programmes, policies or initiatives<br>19. Conclusion and recommendations. List the main conclusions that are justified by the analyses of the data. If appropriate, offer recommendations consistent with a realist approach<br>20. Funding and conflict of interest. State the funding source (if any) for the evaluation, the role played by the funder (if any) and any conflicts of interests of the evaluators.	18<br>18-19<br>19<br>20-21<br>21

Appendix 3: Case study site characteristics at the time of study

Primary care model	Site reference	Hospital size and serving population*	Setting	ED attendances per year	Care Quality Commission (CQC) rating**	GP service commissioning organisation	GP streaming	GP access to acute investigations	Date GP model introduced	Hours of coverage per week in GP service	Staff mix in GP service
No GP service	GPED02 Wales	774 beds, population 600,000 people***	City	84,000	n\a Welsh site						
	GPED12 Central England	350 beds, (Trust serves 500,000 people in 4 hospitals)	Town	65,000	Outstanding (report 04.12.19)						
	GPED15 Central England	497 beds for the 2 hospitals in Trust, population 258,000 people	Town	55,000	Requires improvement (report 29.11.19)						
Inside-integrated model	GPED14 South of England	430 beds, population 450,000 people	Small town	78,000	Outstanding (report 29.04.16)	NHS Trust	No	Yes	2009/10	10am-10pm, 7 days a week; 65 - 72 hours	GPs
	GPED08 North of England	160 beds, population 122,000 people	Rural area	20,000	Good (report 28.10.16)	NHS Trust	No	Yes	2017	8am-6pm, 3-4 days per week; 33 - 40 hours	GPs
	GPED03 South West of England	550 beds, population 340,000 people	Small town in rural area	65,000	Good (report 28.09.17)	NHS Trust	Yes, and GPs self-select	Yes	2017	8am-11pm, 7 days a week; >80 hours	GPs

Inside-parallel model	GPED09 South East of England	995 beds, population 1.3 million people	Large city	165,000	Requires improvement (report 19.07.18)	Primary care provider	Model 1: yes Model 2: no	Model 1: No Model 2: Yes	2012	8am-9pm, 5 days a week; 57 - 64 hours	GPs
	GPED04 North of England	379 beds, population 200,000 people	Rural area	56,000	Good (report 24.02.16)	Primary care provider	Yes	Yes, but encouraged not to use	2015	6am-11pm weekdays, 10am-10pm weekends; 49 - 56 hours	GPs and ANPs
	GPED06 North of England	580 beds, population 200,000 people	Large town	115,000	Good (report 24.02.16)	Primary care provider	Yes, plus 111 and walk in patients	Yes, but encouraged not to use	2015	10am – 10 pm 7 days a week; 80 hours	GPs and ANPs
	GPED07 South West of England	732 beds, population 500,000 people	City	84,000	Requires improvement (report 10.08.16)	Primary care provider until May 2018 then NHS trust	Yes, plus 111 and walk in patients	No	2014	10am – 10pm 7 days a week; 80 hours	GPs, ANPs, Paramedics
Outside-onsite model	GPED13 Central England	540 beds, population 250,000 people	City	70,000	Good (report 29.06.16)	NHS trust	Yes, plus 111 patients (no walk ins)	Yes	2017	10am - 10pm 5 days per week, 41 - 48 hours	GPs and ED ANPs
	GPED10 North of England	572 beds, population 400,000 people	Town	89,000	Good (report 14.03.18)	Local GP federation	Yes, plus 111 and walk in patients	No	2017	24 hours a day (includes OOH contract), 7 days a week; >80 hours	GPs and ANPs
	GPED11 Central England	763 beds, population 514,000 people	Large city hospital adults only	140,000	Requires improvement (report 13.06.16)	NHS trust and a Locum agency	Yes, plus 111 and walk in patients	No	2005	8am-10pm, 7 days per week; 73-80 hours	Locum GPs, mainly ANPs

Data taken from survey data unless stated otherwise *https://www.cqc.org.uk/sites, **for urgent and emergency care services, ***http://www.wales.nhs.uk/sitesplus/866/page/40419
(GPED01 omitted, pilot site; GPED05 omitted, streaming service staffed by emergency department staff not GPs

Appendix 4: Example of realist interview guide – Emergency department staff

Introduce the study to the participant

Thank you for taking part in the interview today. Our aim is to talk to you about you about your role and how the GP-ED model works. We are studying 12 hospitals in England and Wales as part of an NIHR funded project, 9 that have GPs and 3 that do not. We hope to explore what kind of models work in different contexts and what outcomes are achieved. Your interview will form part of our qualitative research evidence and then we will be extracting hospital episode data to look at outcomes such as admissions, use of resources and costs etc.

The interview can last for as long as you are available to speak to me today, please feel free to pause or stop the interview if there is something else you need to deal with. What we talk about during the interview will not be linked to your name as we use ID numbers for all hospitals and staff members that we interview. Please read the patient information and sign the consent form and we can begin the interview when you are ready.

Introductory questions

- I understand that you are an ED doctor and you work alongside GPs?
- How do you feel the GP-ED service works here?

Themes	Questions
**Flow/provider induced demand**	• There's this idea that GPs in the emergency department can improve patient flow **and free up ED staff to deal with sick patients.** Do you have any experience of this? (prompts – individual GP, fills rota gaps, see non urgent patients more quickly, less investigations/admissions<br><br>• There is some suggestion in the literature that patients get to know that there are GPs in the ED and use the service as a **convenient access to primary care, which increases the presentation of patients with primary care problems**, have you any experience of that happening here? (prompts – service not advertised, GPs act as ED staff, rural setting?)
**Role of the GP**	• There's this idea that **GPs may manage patients differently to ED clinicians**, being more comfortable with uncertainty using less investigations and admitting less patients)?<br><br>What is your experience of this? What influences this? Prompts – certain conditions (chest pain/child with fever/abdominal pain)? Different situations? Time of day? Type of patient? Experience of doctor? Because GPs diagnose differently? More comfortable with risk taking? Availability of investigations? Expectation of doing investigations?

**Role of the GP**	• Are there any specific conditions that you **feel GPs manage well or not so well**? (prompts why why why)<br><br>• There's this idea that **GPs may manage patients differently when working in an ED setting utilising more investigations and admitting more patients than they would if they saw the same patient working in the community or OOH**<br>What's your experience of this? (same prompts as above, also personal experience, less knowledge about the patient, expectation to investigate, awareness higher risk of serious illness)
**Safety**	• There is very little in the literature about the **safety implications** about GPs working in or alongside EDs. Are you aware of any positive or negative safety implications?<br>(Prompts positive - primary care patients less over investigation, free up ED staff negative – missed diagnoses, mismanagement sick patients, misinterpretation test results)
**Patient experience**	• There's evidence that when GPs work in an integrated model in EDs, patients are often **not aware that they have seen a GP** and do not report any difference in their experience.<br>Have you any experience of this?
**Team working/ learning/ integration**	• There's this idea that GPs working alongside ED staff **learn from each other** about management pathways in the community and in emergency care which improves the quality care for the patient care<br>Do you have any experience of this? (Prompts – same meetings/protocols/governance/social events/informal conversation?)

Thank you for taking the time to be interviewed today, your responses are valuable to us understanding how GPs work in the department here. Are there any questions that you have or any other comments that you would like to make?

Appendix 5: Marker conditions for patient recruitment

**Marker condition**	**Chief complaint**	**Patient age**	**Exemplar diagnoses**
Child <10 with a fever	Fever	Less than 10	Infectious disease Respiratory Upper respiratory tract infection<br>Surgical ENT Otitis media / ear infection<br>Surgical ENT Tonsillitis
Cough and breathlessness	Short of breath<br>Difficulty breathing<br>Noisy breathing<br>Coughing up blood	Any	Infectious disease Respiratory Lower respiratory tract infection<br>Infectious disease Respiratory Bronchopneumonia<br>Infectious disease Respiratory Lobar pneumonia
Abdominal pain	Abdominal pain	Any	Infectious disease GU / GI Infectious gastroenteritis<br>Infectious disease GU / GI Urinary tract infection
Back pain	Pain in back / trunk (no injury)	Any	Soft tissue injury / wound Muscle injury Lower back<br>Soft tissue injury / wound Sprain / ligament injury Lumbar spine<br>Musculoskeletal Orthopaedics Sciatica
Chest pain	Chest pain	Any	Medical Gastroenterology Oesophageal spasm<br>Medical Gastroenterology Gastro-oesophageal reflux<br>Medical Gastroenterology Gastritis<br>Musculoskeletal Rheumatology Costochondritis<br>Medical Respiratory Pulmonary embolism
Headache	Headache	Any	Migraine<br>Tension Headache<br>Subarachnoid haemorrhage<br>Intracerebral haemorrhage<br>Intracranial space occupying lesion

Cooper A, *et al. Emerg Med J* 2024; 41:287–295. doi: 10.1136/emermed-2023-213426
